# Prof. Coventry’s Introductory Lecture

**Published:** 1850-07

**Authors:** 


*Prof. Coventry's Introductory Lecture.*—We were about writing a notice of this excellent discourse, when the following editorial article in the Boston Medical & Surgical Journal met our eye. As it briefly expresses, in substance, what we designed to express, we take the liberty of appropriating it.

"*Medicine a Science and an Art.*—'A Lecture, introductory to the course in the Medical Institution of Geneva College. By C. B. Coventry, M. D., Professor of Obstetrics, &c. March, 1850. Published by the Class." Prof. Coventry's address is one which would be likely to chain the attention of an audience for the allotted hour, without their knowledge of its having fled. It is one of those spontaneous effusions of a good heart and a cultivated mind, which are well calculated to impress upon the student the most profound devotion to the science in which he is engaged. The profession of medicine is defined most clearly; and they that would have it, '*ars conjecturalis,*' should have heard Dr. C's argument to the contrary. The Dr. asks, 'Can a science which has its foundation in the immutable laws of nature be termed a conjectural art? Does the husbandman, when he sows his seed, merely guess it will grow? Our knowledge is obtained from the well-known, established and immutable laws of nature, as it regards the propagation, growth or development and nourishment of the animal economy; the recorded experience of others for ages, as to the causes and symptoms of disease, and from personal observation and experience of ourselves. Disease arises from the violation of the laws or conditions of health.' 'Scarce is the infant enabled to lisp the name of mother, when what is termed education commences. The brain, still imperfectly organized, is stimulated to unnatural exertion by praise and bribery, and often by exciting some of the worst passions of our nature, rivalry and jealousy.' The innovation in medicine by empirics is illustrated in a most correct and happy manner; and were it not for the press of other matter, we would gladly lay the chapter before our readers."

